# Skull Base Metastasis As the Initial Manifestation of Occult Clear Cell Renal Cell Carcinoma: An Uncommon Cause of Acute Diplopia

**DOI:** 10.7759/cureus.99382

**Published:** 2025-12-16

**Authors:** Sofia Sequeira, Inês Machado Martins, Adriana Santos

**Affiliations:** 1 Internal Medicine, Hospital de Santo Espírito da Ilha Terceira, Angra do Heroísmo, PRT; 2 Nephrology, Hospital de Santo Espírito da Ilha Terceira, Angra do Heroísmo, PRT

**Keywords:** clear cell renal carcinoma, horizontal diplopia, osteolytic bone lesion, paraneoplastic presentation, skull-base metastasis

## Abstract

Skull base metastasis is an uncommon initial presentation of renal cell carcinoma (RCC). Diplopia caused by cranial nerve involvement may be the first and only sign of an otherwise silent malignancy.

A 54-year-old woman presented with sudden horizontal diplopia that resolved with monocular occlusion. Visual acuity and optic nerve examination were normal, yet oculomotor testing showed a right-eye adduction deficit with diplopia on left gaze. Cranial computed tomography (CT) revealed an extensive osteolytic lesion involving multiple structures of the skull base, including the left lateral mass of C1, occipital condyle, foramen magnum margin, jugular foramen, clivus, petrous apex, and sphenoid body. Systemic imaging identified a large heterogeneous right renal mass and widespread osseous metastases. Biopsy confirmed stage IV clear cell RCC. High-dose corticosteroids led to rapid resolution of diplopia, and the patient was referred for systemic oncologic therapy.

Although acute diplopia warrants evaluation for intracranial or skull base pathology, it is rarely the first clinical manifestation of metastatic RCC. Extensive skull base osteolysis should prompt urgent investigation for an underlying malignancy.

## Introduction

Renal cell carcinoma (RCC) accounts for approximately 3% of adult malignancies, with clear cell renal cell carcinoma (ccRCC) being the most common histological subtype [1]. At initial presentation, up to 30% of patients already have metastatic disease, most frequently to the lungs, liver, bone, and brain [1,2]. Although bone metastases are relatively common, skull base involvement remains distinctly rare and is usually described only in isolated case reports [3-6].

Metastatic lesions affecting the clivus, petrous apex, or sphenoid may compress cranial nerves III, IV, or VI within narrow bony canals, resulting in diplopia or isolated cranial neuropathies [3-6]. As RCC often progresses silently and may lack the classic symptoms such as hematuria or flank pain, metastatic manifestations may precede recognition of the primary tumor.

We present a case of occult metastatic ccRCC initially revealed through acute diplopia caused by an extensive skull base lesion. This case highlights the importance of thoroughly evaluating patients with acute diplopia, particularly in the presence of destructive skull base processes, and of maintaining suspicion for metastatic RCC even when systemic symptoms are absent.

## Case presentation

A 54-year-old woman with no significant past medical history presented to the emergency department with sudden-onset horizontal diplopia, which resolved entirely with monocular occlusion. She denied headache, visual loss, facial numbness, dysarthria, dysphagia, limb weakness, fever, night sweats, weight loss, or other systemic symptoms. She remained fully autonomous and cognitively intact.

Ophthalmologic assessment showed preserved bilateral visual acuity and normal optic discs. Oculomotor examination revealed horizontal diplopia on left gaze with a clear right-eye adduction deficit. The remainder of her neurological examination, including cranial nerves, motor function, sensation, and coordination, was normal.

**Figure 1:**
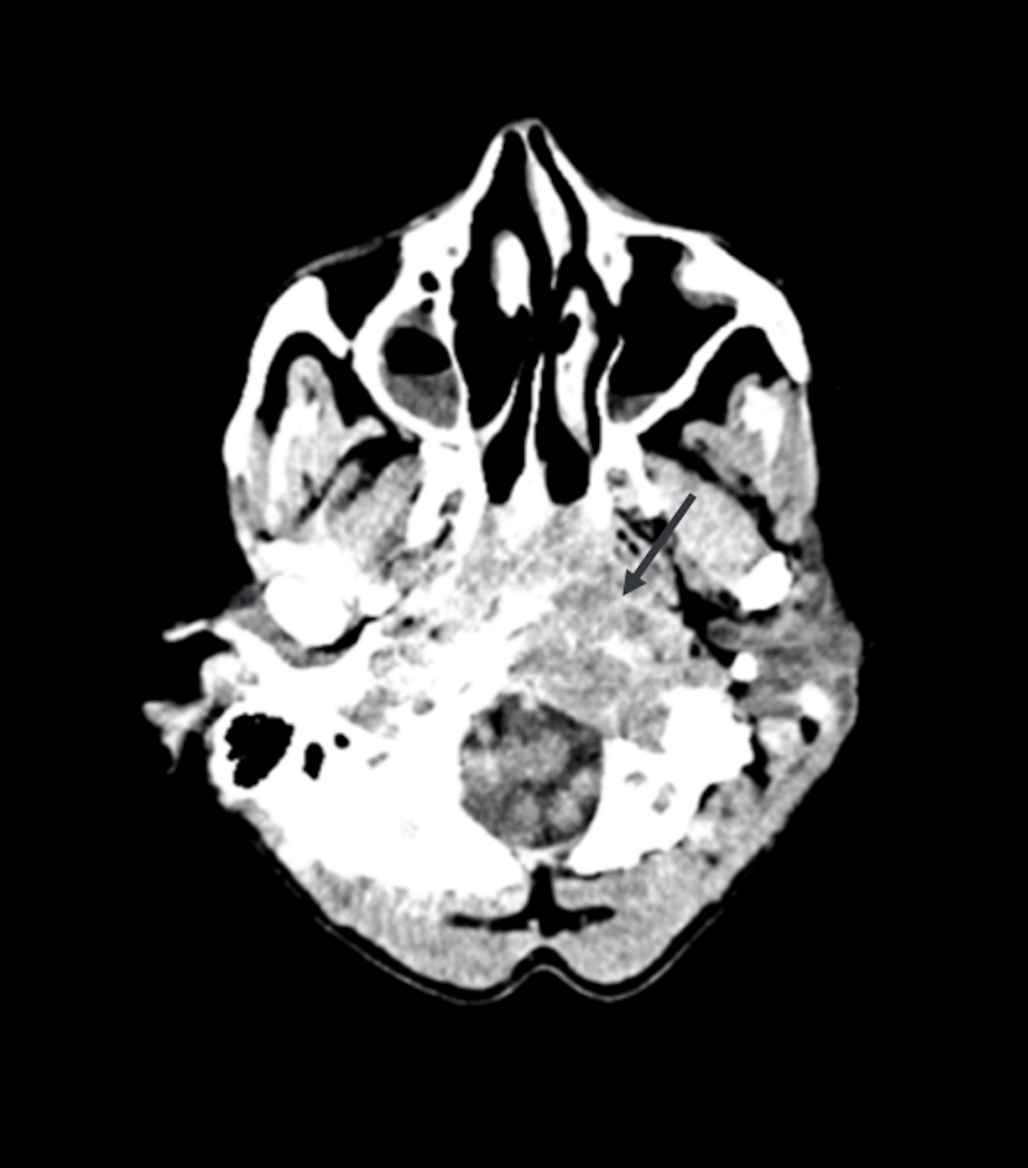
Cranial CT demonstrating extensive osteolysis involving the left occipital condyle and jugular foramen.

A non-contrast cranial CT revealed a large permeative osteolytic lesion involving multiple skull base structures, including the left lateral mass of C1, left occipital condyle, the anterior margin of the foramen magnum, the left jugular foramen, the inferior petrous apex, clivus, and the left sphenoid body (Figure 1). The extent of bone destruction was highly suggestive of metastatic disease.

**Figure 2:**
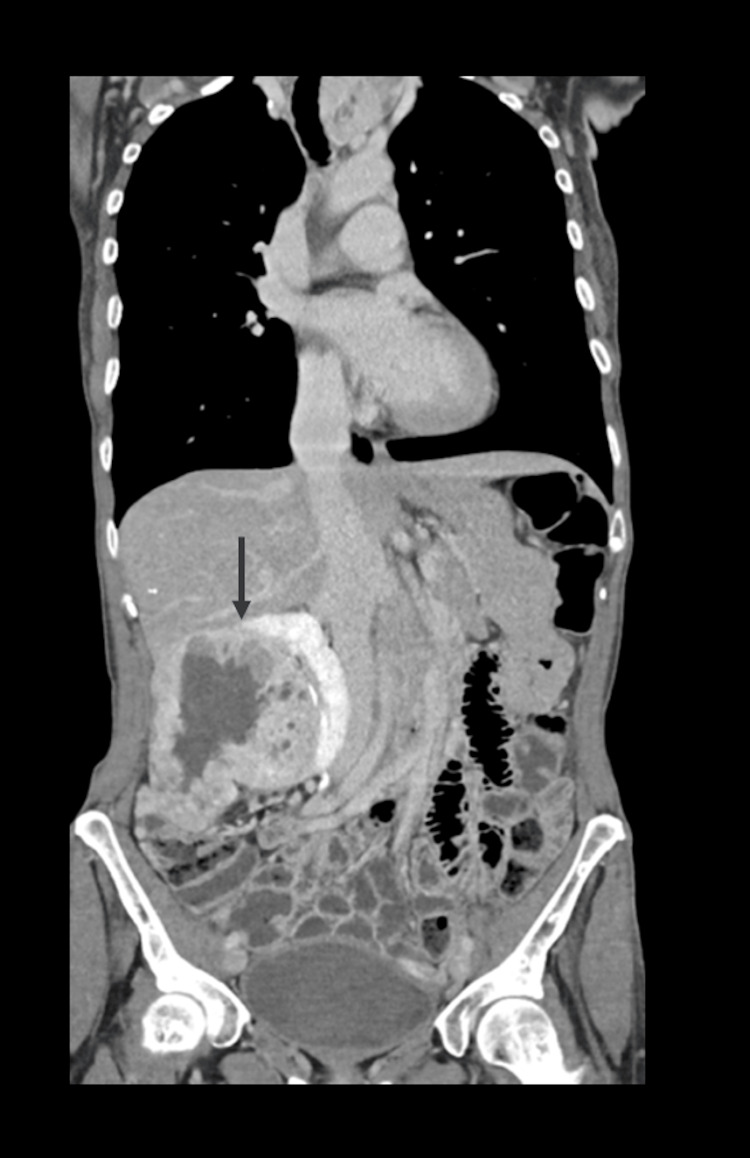
Contrast-enhanced abdominal CT showing a large heterogeneous right renal mass with necrosis and coarse calcifications.

Laboratory testing showed elevated alkaline phosphatase (129 U/L) and mildly increased CA 15-3 (45.5 U/mL), consistent with bone involvement. Contrast-enhanced CT of the chest, abdomen, and pelvis demonstrated a large heterogeneous right renal mass measuring 11.1 × 11.2 × 7.7 cm, with coarse calcifications, necrotic areas, exophytic growth, and loss of the fat plane with the right hepatic lobe. Multiple osteolytic and mixed-density lesions throughout the axial skeleton indicated diffuse metastatic bone disease (Figure 2).

Ultrasound-guided biopsy confirmed clear cell renal cell carcinoma. These findings established the diagnosis of metastatic ccRCC, stage IV.

High-dose intravenous methylprednisolone was initiated to reduce peritumoral edema at the skull base, resulting in rapid and complete resolution of diplopia. The patient was subsequently referred for systemic oncologic therapy.

## Discussion

This case illustrates an uncommon and diagnostically challenging presentation of metastatic ccRCC manifesting exclusively as acute diplopia. Although RCC frequently metastasizes to bone, involvement of the skull base is exceedingly rare and is typically documented only in isolated case reports [3-6]. The clivus, petrous apex, and sphenoid body are anatomically dense regions containing multiple cranial nerves traversing rigid osseous canals; even minimal mass effect or bone destruction in these areas can produce significant neurological deficits.

The patient’s presentation with a right-eye adduction deficit, despite a lesion predominantly affecting the left skull base, highlights the phenomenon of false localizing signs. Similar paradoxical findings have been described in clival and petroclival metastases, where distortion, traction, or angulation of cranial nerves results from displacement of brainstem structures rather than direct involvement of the nerve at its exit point [3,4]. Recognizing this possibility is crucial to avoid misattribution of laterality and to correctly interpret imaging findings.

Renal cell carcinoma often remains clinically silent until advanced stages. Fewer than 10% of patients present with the classical triad of flank pain, hematuria, and a palpable mass, and up to 40% are diagnosed only after metastasis has occurred [1,2]. As demonstrated in this case, neurological symptoms may thus represent the initial manifestation of disease. The absence of constitutional symptoms and the normal ophthalmological examination, apart from restricted ocular motility, underscore the potentially subtle nature of this presentation.

The aggressive osteolytic pattern observed in this patient aligns with the well-recognized biological behavior of RCC metastases, which typically secrete osteoclast-activating cytokines and produce hypervascular lytic lesions [2,7]. The combination of extensive skull base destruction and a large heterogeneous renal mass with necrotic and calcified components strongly suggested metastatic ccRCC, in line with previously reported radiologic patterns [3-6].

The rapid resolution of diplopia following corticosteroid therapy suggests that peritumoral edema substantially contributed to the cranial neuropathy. Although temporary, corticosteroids can stabilize neurological function and provide symptomatic relief while definitive oncologic treatment is planned. Modern systemic therapies - including immune checkpoint inhibitors and vascular endothelial growth factor (VEGF)-targeted agents - have significantly improved outcomes in metastatic RCC, offering meaningful survival benefits compared with historical approaches [2,8]. This case reinforces the importance of considering metastatic RCC in patients presenting with isolated cranial neuropathies and destructive skull base lesions.

## Conclusions

Overall, this case underscores the importance of considering metastatic RCC in the differential diagnosis of destructive skull base lesions and isolated cranial neuropathies, even in the absence of systemic symptoms.

Acute diplopia may represent the first manifestation of advanced disease. When imaging demonstrates aggressive skull base osteolysis, clinicians should promptly evaluate for an underlying malignancy, including RCC. Early recognition facilitates timely oncologic intervention and may help prevent further neurological deterioration.
